# The genome sequence of the Bog Asphodel, *Narthecium ossifragum* (L.) Huds.

**DOI:** 10.12688/wellcomeopenres.24049.1

**Published:** 2025-04-14

**Authors:** Markus Ruhsam, Royal Botanic Garden Edinburgh Genome Acquisition Lab, Darwin Tree of Life Barcoding collective, Plant Genome Sizing collective, Wellcome Sanger Institute Tree of Life Management, Samples and Laboratory team, Wellcome Sanger Institute Scientific Operations: Sequencing Operations, Wellcome Sanger Institute Tree of Life Core Informatics team, Tree of Life Core Informatics collective, Darwin Tree of Life Consortium

**Affiliations:** 1 Royal Botanic Garden Edinburgh, Edinburgh, Scotland, UK

**Keywords:** Narthecium ossifragum, Bog Asphodel, genome sequence, chromosomal, Dioscoreales

## Abstract

We present a genome assembly from a specimen of *Narthecium ossifragum* (Bog Asphodel; Streptophyta; Magnoliopsida; Dioscoreales; Nartheciaceae). The genome sequence has a total length of 378.87 megabases. Most of the assembly (98.91%) is scaffolded into 13 chromosomal pseudomolecules. Two mitochondrial multipartite genomes of 309.19 and 100.78 kilobases, and a plastid genome of 155.31 kilobases were assembled.

## Species taxonomy

Eukaryota; Viridiplantae; Streptophyta; Streptophytina; Embryophyta; Tracheophyta; Euphyllophyta; Spermatophyta; Magnoliopsida; Mesangiospermae; Liliopsida; Petrosaviidae; Dioscoreales; Nartheciaceae; Narthecium; *Narthecium ossifragum* (L.) Huds. (NCBI:txid114204)

## Background

*Narthecium ossifragum* (Bog Asphodel) is a tufted, hairless herbaceous perennial with a creeping rhizome native to western and north-western Europe ( Tutin *et al.*, 1980). It is a light demanding species which grows on acidic and often nutrient-poor substrates like bogs, heaths and moors from sea level to about 1000 m ( Summerfield, 1974). Adult plants are intolerant of shade and gradually disappear when the habitat becomes shaded by taller vegetation ( Tsaliki & Diekmann, 2009). 

The flowers are nectarless but offer pollen rewards and are frequented by a variety of insects ( Jacquemart, 1996; Summerfield, 1974). *Narthecium ossifragum* is a self-compatible species and self-pollination has been shown to occur in the absence of pollinators (fruit set > 90%; Jacquemart, 1996). There are conflicting reports regarding ombrophily in bog asphodel, i.e. the transfer of pollen onto the stigma by rain drops ( Daumann, 1970; Hagerup, 1951; Jacquemart, 1996). The capsules contain numerous tiny seeds that are dispersed either by wind or water ( Summerfield, 1974).

Even though viable seeds are produced every year, they often fail to germinate or show high mortality rates ( Summerfield, 1974; Tsaliki & Diekmann, 2009). It is therefore likely that the often-observed extensive clonal growth in *N. ossifragum* populations is crucial for their persistence ( Summerfield, 1974; Tsaliki & Diekmann, 2009). 

For centuries, bog asphodel has been associated with poisoning of grazing animals ( Paulli, 1667) such as cattle ( Flåøyen *et al.*, 1995), goats ( Wisløff *et al.*, 2003) and sheep ( Ulvund, 2012). For example, photosensitisation has been observed in lambs on pastures containing bog asphodel resulting in lesions on the ears and face with erythema, oedema, ulceration and necrosis that can be followed by secondary infection and death ( Pollock *et al.*, 2015). Although *N. ossifragum* contains hepatotoxic saponins, it is unclear whether the ingestion of this plant is responsible for the observed symptoms as replicating the disease complex by feeding bog asphodel in a controlled environment has been ambiguous ( Pollock *et al.,* 2015). Possible other causes include the intake of toxins from fungal spores ( Di Menna *et al.*, 1992) and cyanobacteria ( Tønnesen *et al.*, 2013).

*Narthecium ossifragum* is a diploid species (2 *n* = 26) ( Hanson *et al.*, 2003; Löve & Löve, 1961; Malmer, 1962).

The genome of the Bog Asphodel, *Narthecium ossifragum*, was sequenced as part of the Darwin Tree of Life Project, a collaborative effort to sequence all named eukaryotic species in the Atlantic Archipelago of Britain and Ireland. Here we present a chromosomally complete genome sequence for *N. ossifragum*, based on a specimen from Glen Coe, United Kingdom.

## Genome sequence report

### Sequencing data

**Figure 1.**
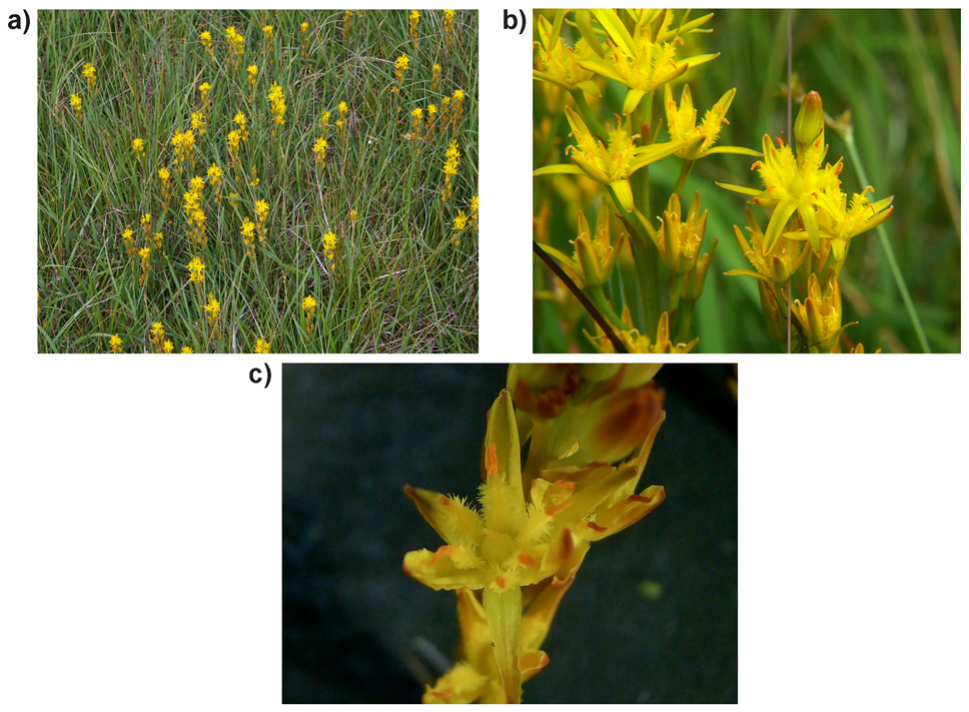
Photographs of the *Narthecium ossifragum* (ldNarOssi1) specimen from which samples were taken for genome sequencing.

The genome of a specimen of *Narthecium ossifragum* ( Figure 1) was sequenced using Pacific Biosciences single-molecule HiFi long reads, generating 28.14 Gb (gigabases) from 2.13 million reads. GenomeScope analysis of the PacBio HiFi data estimated the haploid genome size at 346.48 Mb, with a heterozygosity of 0.60% and repeat content of 45.68%. Based on this estimated genome size, the sequencing data provided approximately 71.0x coverage of the genome. Using flow cytometry, the genome size (1C-value) was estimated to be 0.51 pg, equivalent to 490 Mb. Chromosome conformation Hi-C sequencing produced 235.70 Gb from 1,560.93 million reads. Table 1 summarises the specimen and sequencing information.

**Table 1.** Specimen and sequencing data for *Narthecium ossifragum*.

Project information			
**Study title**	Narthecium ossifragum		
**Umbrella BioProject**	PRJEB65688		
**Species**	*Narthecium ossifragum*		
**BioSpecimen**	SAMEA10983567		
**NCBI taxonomy ID**	114204		
Specimen information			
**Technology**	**ToLID**	**BioSample accession**	**Organism part**
**PacBio long read sequencing**	ldNarOssi1	SAMEA10983678	leaf
**Hi-C sequencing**	ldNarOssi1	SAMEA10983678	leaf
Sequencing information			
**Platform**	**Run accession**	**Read count**	**Base count (Gb)**
**Hi-C Illumina NovaSeq 6000**	ERR12035270	7.20e+08	108.7
**Hi-C Illumina NovaSeq 6000**	ERR12035269	8.41e+08	127.0
**PacBio Sequel IIe**	ERR12015745	2.13e+06	28.14

### Assembly statistics

The primary haplotype was assembled, and contigs corresponding to an alternate haplotype were also deposited in INSDC databases. The assembly was improved by manual curation, which corrected 378 misjoins or missing joins and removed 7 haplotypic duplications. These interventions decreased the scaffold count by 46.28% and decreased the scaffold N50 by 8.18%. The final assembly has a total length of 378.87 Mb in 156 scaffolds, with 2,687 gaps, and a scaffold N50 of 28.57 Mb ( Table 2).

**Table 2.** Genome assembly data for *Narthecium ossifragum*.

Genome assembly		
Assembly name	ldNarOssi1.1	
Assembly accession	GCA_963920575.1	
*Alternate haplotype accession*	*GCA_963920555.1*	
Assembly level for primary assembly	chromosome	
Span (Mb)	378.87	
Number of contigs	2,843	
Number of scaffolds	156	
Longest scaffold (Mb)	39.38	
Assembly metric	Measure	*Benchmark*
Contig N50 length	0.21 Mb	*≥ 1 Mb*
Scaffold N50 length	28.57 Mb	*= chromosome N50*
Consensus quality (QV)	Primary: 60.5; alternate: 55.2; combined: 59.1	*≥ 40*
*k*-mer completeness	Primary: 98.20%; alternate: 17.93%; combined: 98.78%	*≥ 95%*
BUSCO *	C:87.9%[S:85.5%,D:2.4%], F:7.0%,M:5.1%,n:3,236	*S > 90%; D < 5%*
Percentage of assembly mapped to chromosomes	98.91%	*≥ 90%*
Organelles	Mitochondrial genomes: 309.19 and 100.78 kb, Plastid genome: 155.31 kb	*complete single alleles*

* BUSCO scores based on the BUSCO set using version 5.5.0. C = complete [S = single copy, D = duplicated], F = fragmented, M = missing, n = number of orthologues in comparison.

**Figure 2.**
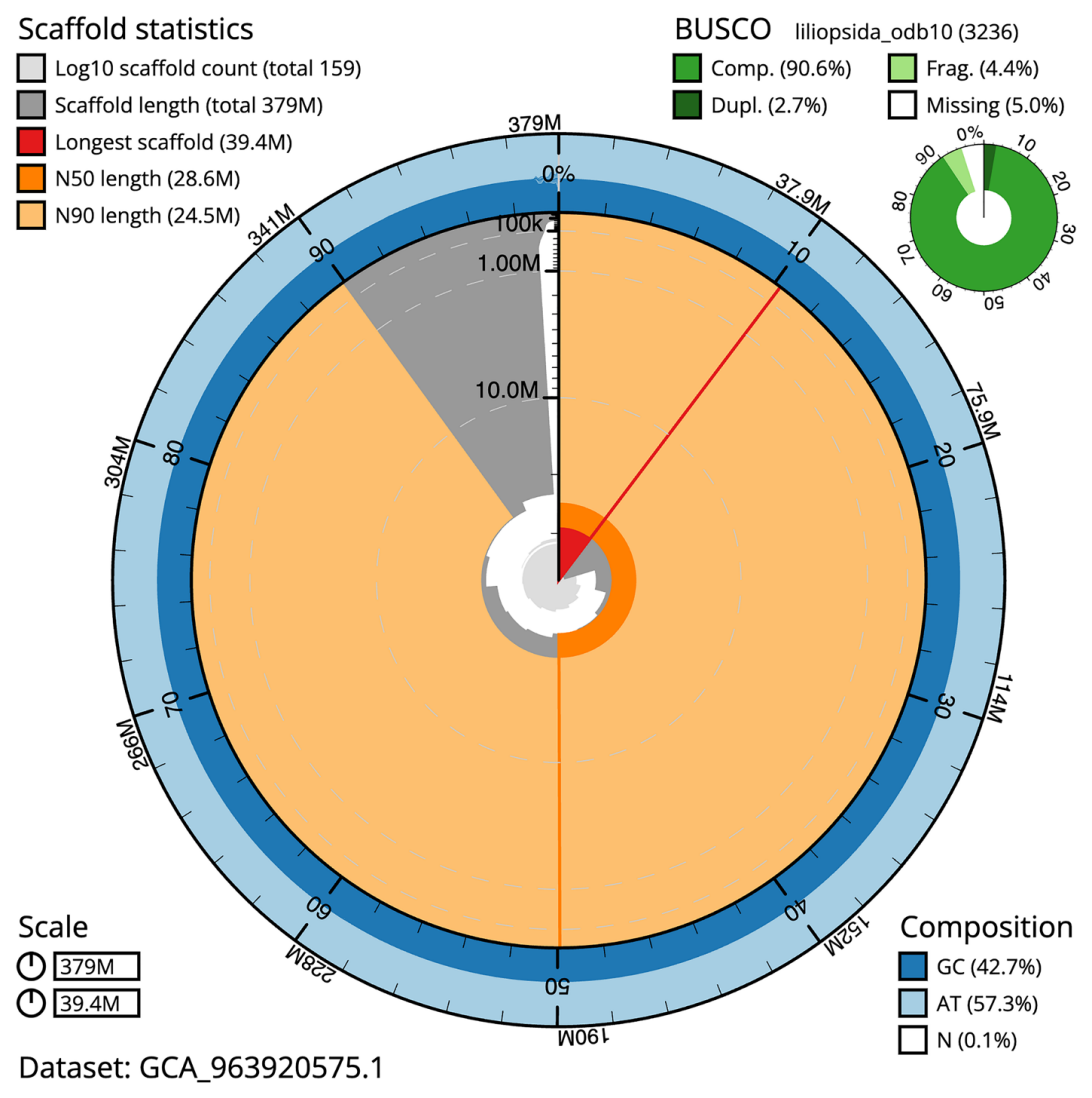
Snail plot summary of assembly statistics for assembly ldNarOssi1.1: metrics. The BlobToolKit snail plot provides an overview of assembly metrics and BUSCO gene completeness. The circumference represents the length of the whole genome sequence, and the main plot is divided into 1,000 bins around the circumference. The outermost blue tracks display the distribution of GC, AT, and N percentages across the bins. Scaffolds are arranged clockwise from longest to shortest and are depicted in dark grey. The longest scaffold is indicated by the red arc, and the deeper orange and pale orange arcs represent the N50 and N90 lengths. A light grey spiral at the centre shows the cumulative scaffold count on a logarithmic scale. A summary of complete, fragmented, duplicated, and missing BUSCO genes in the liliopsida_odb10 set is presented at the top right. An interactive version of this figure is available at https://blobtoolkit.genomehubs.org/view/GCA_963920575.1/dataset/GCA_963920575.1/snail.

**Figure 3.**
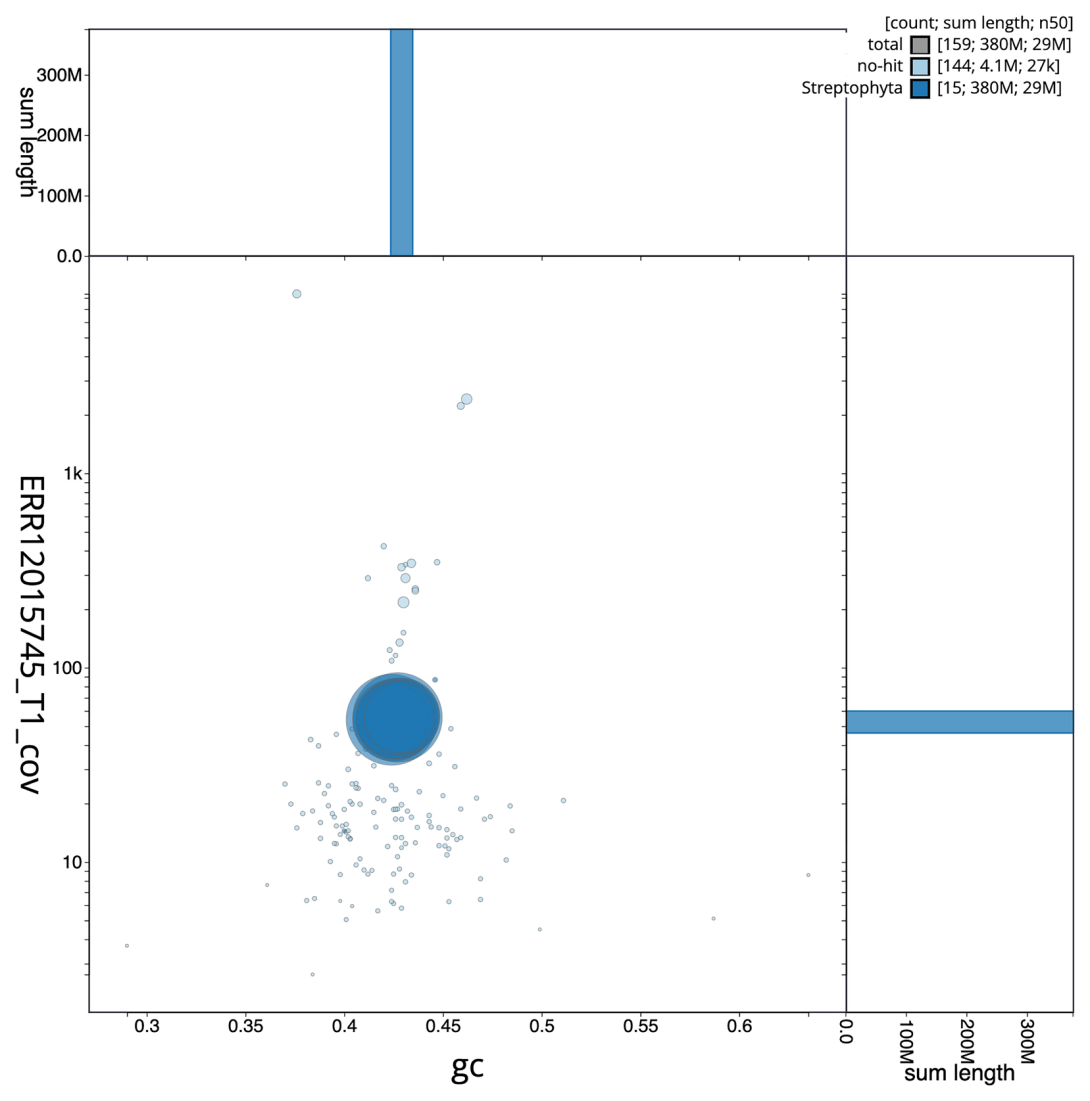
BlobToolKit blob plot for assembly ldNarOssi1.1: BlobToolKit GC-coverage plot showing sequence coverage (vertical axis) and GC content (horizontal axis). The circles represent scaffolds, with the size proportional to scaffold length and the colour representing phylum membership. The histograms along the axes display the total length of sequences distributed across different levels of coverage and GC content. An interactive version of this figure is available at https://blobtoolkit.genomehubs.org/view/GCA_963920575.1/dataset/GCA_963920575.1/blob.

**Figure 4.**
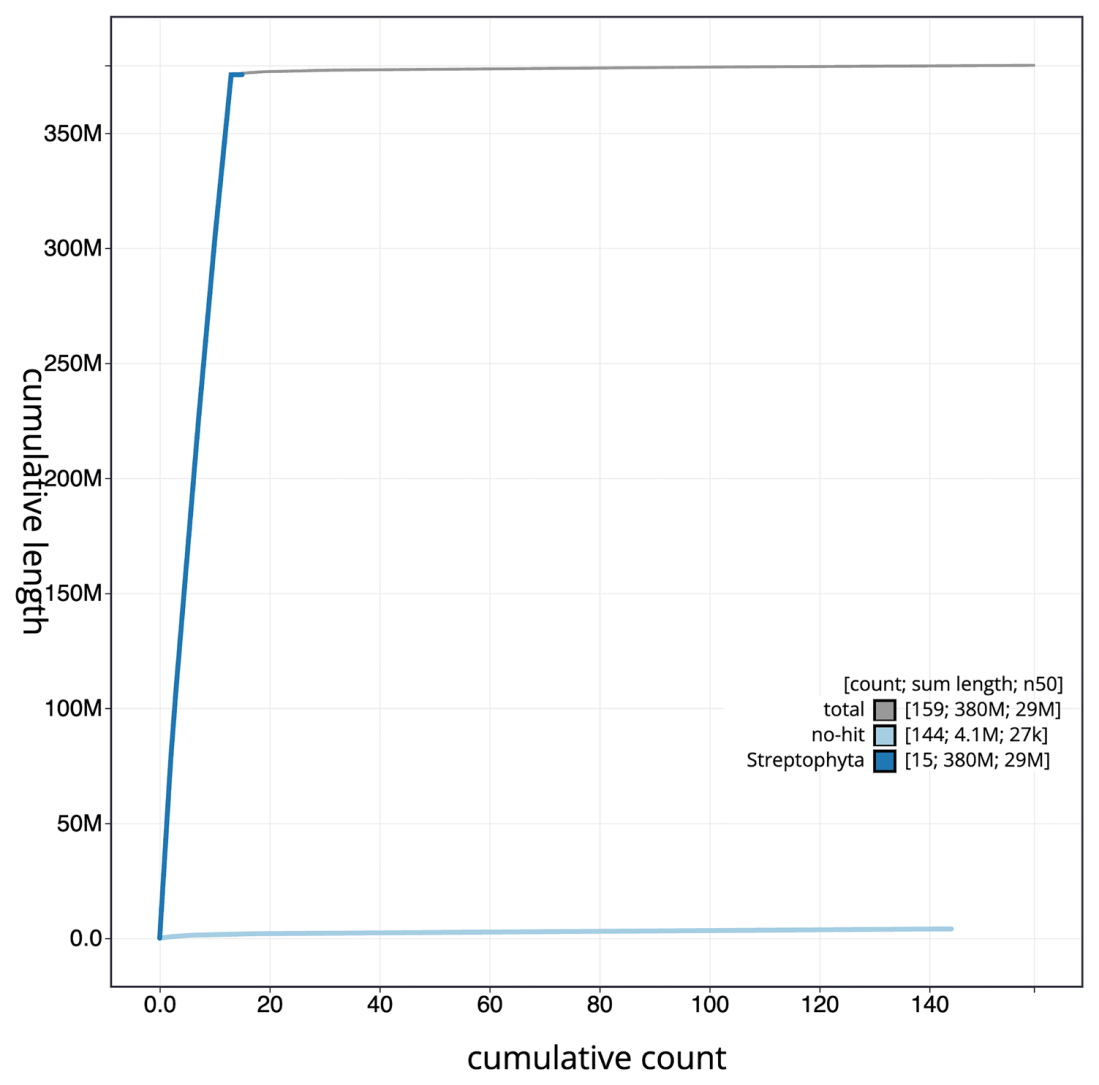
BlobToolKit cumulative sequence plot. The grey line shows cumulative length for all scaffolds. Coloured lines show cumulative lengths of scaffolds assigned to each phylum using the buscogenes taxrule. An interactive version of this figure is available at https://blobtoolkit.genomehubs.org/view/GCA_963920575.1/dataset/GCA_963920575.1/cumulative.

The snail plot in Figure 2 provides a summary of the assembly statistics, indicating the distribution of scaffold lengths and other assembly metrics. Figure 3 shows the distribution of scaffolds by GC proportion and coverage. Figure 4 presents a cumulative assembly plot, with separate curves representing different scaffold subsets assigned to various phyla, illustrating the completeness of the assembly.

**Figure 5.**
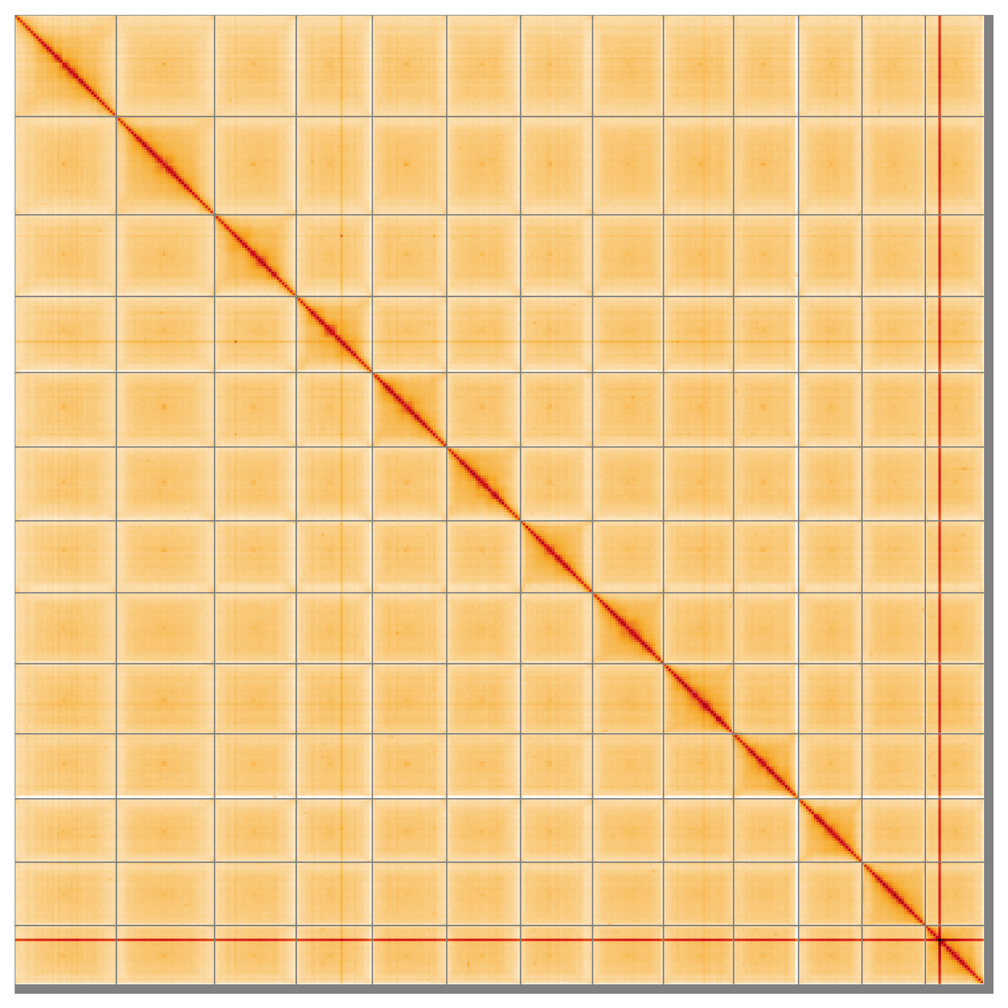
Genome assembly of *Narthecium ossifragum*, ldNarOssi1.1: Hi-C contact map of the ldNarOssi1.1 assembly, visualised using HiGlass. Chromosomes are shown in order of size from left to right and top to bottom. Darker shades indicate more frequent physical contacts between regions, while lighter areas represent fewer contacts. An interactive version of this figure may be viewed at https://genome-note-higlass.tol.sanger.ac.uk/l/?d=RWrcqJKqQA2xxgpvTN5KjA.

Most of the assembly sequence (99.07%) was assigned to 13 chromosomal-level scaffolds. These chromosome-level scaffolds, confirmed by Hi-C data, are named according to size ( Figure 5; Table 3).

**Table 3.** Chromosomal pseudomolecules in the genome assembly of *Narthecium ossifragum*, ldNarOssi1.

INSDC accession	Name	Length (Mb)	GC%
OY986963.1	1	39.38	42.5
OY986964.1	2	37.98	42.5
OY986965.1	3	31.6	42.5
OY986966.1	4	29.44	42.5
OY986967.1	5	28.81	42.5
OY986968.1	6	28.57	42.5
OY986969.1	7	27.83	42.5
OY986970.1	8	27.46	43
OY986971.1	9	27.1	43
OY986972.1	10	25.16	43
OY986973.1	11	24.54	42.5
OY986974.1	12	24.5	42.5
OY986975.1	13	22.97	43
OY986978.1	Pltd	0.16	37.5
OY986976.1	MT1	0.31	46
OY986977.1	MT2	0.1	46

The mitochondrial and plastid genomes were also assembled. These sequences are included as contigs in the multifasta file of the genome submission and as standalone records in GenBank.

### Assembly quality metrics

The estimated Quality Value (QV) and *k*-mer completeness metrics, along with BUSCO completeness scores, were calculated for each haplotype and the combined assembly. The QV reflects the base-level accuracy of the assembly, while *k*-mer completeness indicates the proportion of expected *k*-mers identified in the assembly. BUSCO scores provide a measure of completeness based on benchmarking universal single-copy orthologues.

The primary haplotype has a QV of 60.5, and the combined primary and alternate assemblies achieve an estimated QV of 59.1. The *k*-mer recovery for the primary haplotype is 98.20%, and for the alternate haplotype 17.93%; the combined primary and alternate assemblies have a *k*-mer recovery of 98.78%. The primary assembly has a BUSCO completeness of 87.9% (single = 85.5%, duplicated = 2.4%), using the liliopsida_odb10 reference set ( *n* = 3,236).

Table 2 provides assembly metric benchmarks adapted from Rhie *et al.* (2021) and the Earth BioGenome Project Report (EBP) on Assembly Standards September 2024. The assembly achieves the EBP reference standard of **5.C.Q60**.

## Methods

### Sample acquisition, DNA barcoding and genome size estimation

A specimen of *Narthecium ossifragum* (specimen ID EDTOL01473, ToLID ldNarOssi1) was collected from Glen Coe, United Kingdom (latitude 56.66, longitude –5.07) on 2021-06-22. The specimen was collected and identified by Markus Ruhsam (Royal Botanic Garden Edinburgh) and preserved inliquid nitrogen. The herbarium voucher associated with the sequenced plant is https://data.rbge.org.uk/herb/E01152278 and is deposited in the herbarium of RBG Edinburgh (E). Metadata collection for samples adhered to the Darwin Tree of Life project standards described by Lawniczak *et al*. (2022).

The initial species identification was verified by an additional DNA barcoding process according to the framework developed by Twyford *et al*. (2024). Part of the plant specimen was preserved in silica gel desiccant ( Chase & Hills, 1991). A DNA extraction from the dried plant was amplified by PCR for standard barcode markers, with the amplicons sequenced and compared to public sequence databases including GenBank and the Barcode of Life Database (BOLD). The barcode sequences for this specimen are openly available on BOLD ( Ratnasingham & Hebert, 2007). Following whole genome sequence generation, DNA barcodes were also used alongside the initial barcoding data for sample tracking through the genome production pipeline at the Wellcome Sanger Institute ( Twyford *et al.*, 2024). The standard operating procedures for the Darwin Tree of Life barcoding have been deposited on protocols.io ( Beasley *et al.*, 2023).

The genome size was estimated by flow cytometry using the fluorochrome propidium iodide and following the ‘one-step’ method as outlined in Pellicer *et al.* (2021). For this species, the General Purpose Buffer (GPB) supplemented with 3% PVP and 0.08% (v/v) beta-mercaptoethanol was used for isolation of nuclei ( Loureiro *et al.*, 2007), and the internal calibration standard was *Solanum lycopersicum* ‘Stupiké polní rané’ with an assumed 1C-value of 968 Mb ( Doležel *et al.*, 2007).

### Nucleic acid extraction

The workflow for high molecular weight (HMW) DNA extraction at the WSI Tree of Life Core Laboratory includes a sequence of core procedures: sample preparation and homogenisation, DNA extraction, fragmentation and purification. Detailed protocols are available on protocols.io ( Denton *et al.*, 2023). The ldNarOssi1 sample was weighed and dissected on dry ice ( Jay *et al.*, 2023). For sample homogenisation, leaf tissue was cryogenically disrupted using the Covaris cryoPREP ^®^ Automated Dry Pulverizer ( Narváez-Gómez *et al.*, 2023).

HMW DNA was extracted using the Automated Plant MagAttract v2 protocol ( Todorovic *et al.*, 2023). For ultra-low input (ULI) PacBio sequencing, DNA was fragmented using the Covaris g-TUBE method ( Oatley *et al.*, 2023). Sheared DNA was purified by solid-phase reversible immobilisation, using AMPure PB beads to eliminate shorter fragments and concentrate the DNA ( Strickland *et al.*, 2023). The concentration of the sheared and purified DNA was assessed using a Nanodrop spectrophotometer and Qubit Fluorometer and Qubit dsDNA High Sensitivity Assay kit. Fragment size distribution was evaluated by running the sample on the FemtoPulse system.

### Hi-C sample preparation and crosslinking

Hi-C data were generated from the leaf tissue of the ldNarOssi1 sample using the Arima-HiC v2 kit (Arima Genomics). Tissue was finely ground using the Covaris cryoPREP Dry Pulverizer (Covaris) and then subjected to nuclei isolation. Nuclei were isolated using a modified protocol of the Qiagen QProteome Cell Compartment Kit (Qiagen) where only Lysis and CE2 buffers are used with QiaShredder spin columns. After isolation, nuclei were fixed using formaldehyde solution with a final concentration of 2% to crosslink the DNA. The crosslinked DNA was then digested and biotinylated per the manufacturer's instructions. A clean up was performed with SPRIselect beads prior to library preparation. DNA concentration was quantified using the Qubit Fluorometer v4.0 (Thermo Fisher Scientific) and Qubit HS Assay Kit according to the manufacturer’s instructions.

### Library preparation and sequencing

Library preparation and sequencing were performed at the WSI Scientific Operations core.

***PacBio HiFi***

The sample requires Covaris g-TUBE shearing to approximately 10 kb prior to library preparation. Ultra-low input libraries were prepared using PacBio SMRTbell® Express Template Prep Kit 2.0 and PacBio SMRTbell® gDNA Sample Amplification Kit. To begin, samples were normalised to 20 ng of DNA. Initial removal of single-strand overhangs, DNA damage repair, and end repair/A-tailing were performed per manufacturer’s instructions. From the SMRTbell® gDNA Sample Amplification Kit, amplification adapters were then ligated. A 0.85X pre-PCR clean-up was performed with Promega ProNex beads and the sample was then divided into two for a dual PCR. PCR reactions A and B each followed the PCR programs as described in the manufacturer’s protocol. A 0.85X post-PCR clean-up was performed with ProNex beads for PCR reactions A and B and DNA concentration was quantified using the Qubit Fluorometer v4.0 (Thermo Fisher Scientific) and Qubit HS Assay Kit and fragment size analysis was carried out using the Agilent Femto Pulse Automated Pulsed Field CE Instrument (Agilent Technologies) and gDNA 55kb BAC analysis kit. PCR reactions A and B were then pooled, ensuring the total mass was ≥500 ng in 47.4 μl. The pooled sample then repeated the process for DNA damage repair, end repair/A-tailing and additional hairpin adapter ligation. A 1X clean-up was performed with ProNex beads and DNA concentration was quantified using the Qubit and fragment size analysis was carried out using the Agilent Femto Pulse Automated Pulsed Field CE Instrument (Agilent Technologies). Size selection was performed using Sage Sciences' PippinHT system with target fragment size determined by analysis from the Femto Pulse, usually a value between 4000 and 9000 bp. Size selected libraries were then cleaned-up using1.0X ProNex beads and normalised to 2 nM before proceeding to sequencing.

Samples were sequenced on a Sequel IIe instrument (Pacific Biosciences, California, USA). The concentration of the library loaded onto the Sequel IIe was in the range 40–135 pM. The SMRT link software, a PacBio web-based end-to-end workflow manager, was used to set-up and monitor the run, as well as perform primary and secondary analysis of the data upon completion.

***Hi-C***

For Hi-C library preparation, DNA was fragmented to a size of 400 to 600 bp using a Covaris E220 sonicator. The DNA was then enriched, barcoded, and amplified using the NEBNext Ultra II DNA Library Prep Kit (New England Biolabs) following manufacturer’s instructions. Prior to sequencing, libraries were normalised to 10 ng/uL. Hi-C sequencing was performed using paired-end sequencing with a read length of 150 bp on an Illumina NovaSeq 6000 instrument.

### Genome assembly, curation and evaluation

***Assembly***

Prior to assembly of the PacBio HiFi reads, a database of *k*-mer counts ( *k* = 31) was generated from the filtered reads using FastK. GenomeScope2 ( Ranallo-Benavidez *et al.*, 2020) was used to analyse the *k*-mer frequency distributions, providing estimates of genome size, heterozygosity, and repeat content.

The HiFi reads were assembled using Hifiasm in Hi-C phasing mode ( Cheng *et al.*, 2021; Cheng *et al.*, 2022), resulting in a pair of haplotype-resolved assemblies. The Hi-C reads were mapped to the primary contigs using bwa-mem2 ( Vasimuddin *et al.*, 2019). The contigs were further scaffolded using the provided Hi-C data ( Rao *et al.*, 2014) in YaHS ( Zhou *et al.*, 2023) using the --break option for handling potential misassemblies. The scaffolded assemblies were evaluated using Gfastats ( Formenti *et al.*, 2022), BUSCO ( Manni *et al.*, 2021) and MERQURY.FK ( Rhie *et al.*, 2020).

The organelle genomes were assembled using MitoHiFi ( Uliano-Silva *et al.*, 2023) and OATK ( Zhou, 2023).

***Assembly curation***

The assembly was decontaminated using the Assembly Screen for Cobionts and Contaminants (ASCC) pipeline. Flat files and maps used in curation were generated via the TreeVal pipeline ( Pointon *et al.*, 2023). Manual curation was conducted primarily in PretextView ( Harry, 2022) and HiGlass ( Kerpedjiev *et al.*, 2018), with additional insights provided by JBrowse2 ( Diesh *et al.*, 2023). Scaffolds were visually inspected and corrected as described by Howe *et al*. (2021). Any identified contamination, missed joins, and mis-joins were amended, and duplicate sequences were tagged and removed. The curation process is documented at https://gitlab.com/wtsi-grit/rapid-curation.

***Evaluation of assembly quality***

The Merqury.FK tool ( Rhie *et al.*, 2020), run in a Singularity container ( Kurtzer *et al.*, 2017), was used to evaluate *k*-mer completeness and assembly quality for the primary and alternate haplotypes using the *k*-mer databases ( *k* = 31) computed prior to genome assembly. The analysis outputs included assembly QV scores and completeness statistics.

A Hi-C contact map was produced for the final version of the assembly. The Hi-C reads were aligned using bwa-mem2 ( Vasimuddin *et al.*, 2019) and the alignment files were combined using SAMtools ( Danecek *et al.*, 2021). The Hi-C alignments were converted into a contact map using BEDTools ( Quinlan & Hall, 2010) and the Cooler tool suite ( Abdennur & Mirny, 2020). The contact map was visualised in HiGlass ( Kerpedjiev *et al.*, 2018).

The blobtoolkit pipeline is a Nextflow port of the previous Snakemake Blobtoolkit pipeline ( Challis *et al.*, 2020). It aligns the PacBio reads in SAMtools and minimap2 ( Li, 2018) and generates coverage tracks for regions of fixed size. In parallel, it queries the GoaT database ( Challis *et al.*, 2023) to identify all matching BUSCO lineages to run BUSCO ( Manni *et al.*, 2021). For the three domain-level BUSCO lineages, the pipeline aligns the BUSCO genes to the UniProt Reference Proteomes database ( Bateman *et al.*, 2023) with DIAMOND ( Buchfink *et al.*, 2021) blastp. The genome is also split into chunks according to the density of the BUSCO genes from the closest taxonomic lineage, and each chunk is aligned to the UniProt Reference Proteomes database with DIAMOND blastx. Genome sequences with no hits are chunked with seqtk and aligned to the NT database with blastn ( Altschul *et al.*, 1990). The blobtools suite combines all these outputs into a blobdir for visualisation.

The blobtoolkit pipeline was developed using nf-core tooling ( Ewels *et al.*, 2020) and MultiQC ( Ewels *et al.*, 2016), relying on the Conda package manager, the Bioconda initiative ( Grüning *et al.*, 2018), the Biocontainers infrastructure ( da Veiga Leprevost *et al.*, 2017), as well as the Docker ( Merkel, 2014) and Singularity ( Kurtzer *et al.*, 2017) containerisation solutions.

Table 4 contains a list of relevant software tool versions and sources.

**Table 4.** Software tools: versions and sources.

Software tool	Version	Source
BEDTools	2.30.0	https://github.com/arq5x/bedtools2
BLAST	2.14.0	ftp://ftp.ncbi.nlm.nih.gov/blast/executables/blast+/
BlobToolKit	4.3.3	https://github.com/blobtoolkit/blobtoolkit
BUSCO	5.5.0	https://gitlab.com/ezlab/busco
bwa-mem2	2.2.1	https://github.com/bwa-mem2/bwa-mem2
Cooler	0.8.11	https://github.com/open2c/cooler
DIAMOND	2.1.8	https://github.com/bbuchfink/diamond
fasta_windows	0.2.4	https://github.com/tolkit/fasta_windows
FastK	427104ea91c78c3b8b8b49f1a7d6bbeaa869ba1c	https://github.com/thegenemyers/FASTK
Gfastats	1.3.6	https://github.com/vgl-hub/gfastats
GoaT CLI	0.2.5	https://github.com/genomehubs/goat-cli
Hifiasm	0.16.1-r375	https://github.com/chhylp123/hifiasm
HiGlass	44086069ee7d4d3f6f3f0012569789ec138f42b84 aa44357826c0b6753eb28de	https://github.com/higlass/higlass
MerquryFK	d00d98157618f4e8d1a9190026b19b471055b22e	https://github.com/thegenemyers/MERQURY.FK
Minimap2	2.24-r1122	https://github.com/lh3/minimap2
MitoHiFi	None	https://github.com/marcelauliano/MitoHiFi
MultiQC	1.14, 1.17, and 1.18	https://github.com/MultiQC/MultiQC
NCBI Datasets	15.12.0	https://github.com/ncbi/datasets
Nextflow	23.04.1	https://github.com/nextflow-io/nextflow
OATK	0.2	https://github.com/c-zhou/oatk
PretextView	0.2.5	https://github.com/sanger-tol/PretextView
samtools	1.18	https://github.com/samtools/samtools
sanger-tol/ascc	-	https://github.com/sanger-tol/ascc
sanger-tol/blobtoolkit	0.5.1	https://github.com/sanger-tol/blobtoolkit
Seqtk	1.3	https://github.com/lh3/seqtk
Singularity	3.9.0	https://github.com/sylabs/singularity
TreeVal	1.2.0	https://github.com/sanger-tol/treeval
YaHS	1.1a.2	https://github.com/c-zhou/yahs

### Wellcome Sanger Institute – Legal and Governance

The materials that have contributed to this genome note have been supplied by a Darwin Tree of Life Partner. The submission of materials by a Darwin Tree of Life Partner is subject to the **‘Darwin Tree of Life Project Sampling Code of Practice’**, which can be found in full on the Darwin Tree of Life website here. By agreeing with and signing up to the Sampling Code of Practice, the Darwin Tree of Life Partner agrees they will meet the legal and ethical requirements and standards set out within this document in respect of all samples acquired for, and supplied to, the Darwin Tree of Life Project. 

Further, the Wellcome Sanger Institute employs a process whereby due diligence is carried out proportionate to the nature of the materials themselves, and the circumstances under which they have been/are to be collected and provided for use. The purpose of this is to address and mitigate any potential legal and/or ethical implications of receipt and use of the materials as part of the research project, and to ensure that in doing so we align with best practice wherever possible. The overarching areas of consideration are:

• Ethical review of provenance and sourcing of the material

• Legality of collection, transfer and use (national and international) 

Each transfer of samples is further undertaken according to a Research Collaboration Agreement or Material Transfer Agreement entered into by the Darwin Tree of Life Partner, Genome Research Limited (operating as the Wellcome Sanger Institute), and in some circumstances other Darwin Tree of Life collaborators.

## Data Availability

European Nucleotide Archive: Narthecium ossifragum. Accession number PRJEB65688; https://identifiers.org/ena.embl/PRJEB65688. The genome sequence is released openly for reuse. The *Narthecium ossifragum* genome sequencing initiative is part of the Darwin Tree of Life (DToL) project. All raw sequence data and the assembly have been deposited in INSDC databases. The genome will be annotated using available RNA-Seq data and presented through the Ensembl pipeline at the European Bioinformatics Institute. Raw data and assembly accession identifiers are reported in Table 1.

## Author information

Members of the Royal Botanic Garden Edinburgh Genome Acquisition Lab are listed here: https://doi.org/10.5281/zenodo.4786682.

Members of the Plant Genome Sizing collective are listed here: https://doi.org/10.5281/zenodo.7994306.

Members of the Darwin Tree of Life Barcoding collective are listed here: https://doi.org/10.5281/zenodo.12158331

Members of the Wellcome Sanger Institute Tree of Life Management, Samples and Laboratory team are listed here: https://doi.org/10.5281/zenodo.12162482.

Members of Wellcome Sanger Institute Scientific Operations: Sequencing Operations are listed here: https://doi.org/10.5281/zenodo.12165051.

Members of the Wellcome Sanger Institute Tree of Life Core Informatics team are listed here: https://doi.org/10.5281/zenodo.12160324.

Members of the Tree of Life Core Informatics collective are listed here: https://doi.org/10.5281/zenodo.12205391.

Members of the Darwin Tree of Life Consortium are listed here: https://doi.org/10.5281/zenodo.4783558.

## References

[ref-1] Abdennur N Mirny LA : Cooler: scalable storage for Hi-C data and other genomically labeled arrays. *Bioinformatics.* 2020;36(1):311–316. 10.1093/bioinformatics/btz540 31290943 PMC8205516

[ref-2] Altschul SF Gish W Miller W : Basic Local Alignment Search Tool. *J Mol Biol.* 1990;215(3):403–410. 10.1016/S0022-2836(05)80360-2 2231712

[ref-3] Bateman A Martin MJ Orchard S : UniProt: the universal protein knowledgebase in 2023. *Nucleic Acids Res.* 2023;51(D1):D523–D531. 10.1093/nar/gkac1052 36408920 PMC9825514

[ref-4] Beasley J Uhl R Forrest LL : DNA barcoding SOPs for the Darwin Tree of Life project. *protocols.io.* 2023; [Accessed 25 June 2024]. 10.17504/protocols.io.261ged91jv47/v1

[ref-5] Buchfink B Reuter K Drost HG : Sensitive protein alignments at Tree-of-Life scale using DIAMOND. *Nat Methods.* 2021;18(4):366–368. 10.1038/s41592-021-01101-x 33828273 PMC8026399

[ref-6] Challis R Kumar S Sotero-Caio C : Genomes on a Tree (GoaT): a versatile, scalable search engine for genomic and sequencing project metadata across the eukaryotic Tree of Life [version 1; peer review: 2 approved]. *Wellcome Open Res.* 2023;8:24. 10.12688/wellcomeopenres.18658.1 36864925 PMC9971660

[ref-7] Challis R Richards E Rajan J : BlobToolKit – interactive quality assessment of genome assemblies. *G3 (Bethesda).* 2020;10(4):1361–1374. 10.1534/g3.119.400908 32071071 PMC7144090

[ref-8] Chase MW Hills HH : Silica gel: an ideal material for field preservation of leaf samples for DNA studies. *Taxon.* 1991;40(2):215–220. 10.2307/1222975

[ref-9] Cheng H Concepcion GT Feng X : Haplotype-resolved *de novo* assembly using phased assembly graphs with hifiasm. *Nat Methods.* 2021;18(2):170–175. 10.1038/s41592-020-01056-5 33526886 PMC7961889

[ref-10] Cheng H Jarvis ED Fedrigo O : Haplotype-resolved assembly of diploid genomes without parental data. *Nat Biotechnol.* 2022;40(9):1332–1335. 10.1038/s41587-022-01261-x 35332338 PMC9464699

[ref-11] da Veiga Leprevost F Grüning BA Alves Aflitos S : BioContainers: an open-source and community-driven framework for software standardization. *Bioinformatics.* 2017;33(16):2580–2582. 10.1093/bioinformatics/btx192 28379341 PMC5870671

[ref-12] Danecek P Bonfield JK Liddle J : Twelve years of SAMtools and BCFtools. *GigaScience.* 2021;10(2): giab008. 10.1093/gigascience/giab008 33590861 PMC7931819

[ref-13] Daumann E : Zur Frage nach der Bestäubung Durch Regen (Ombrogamie). *Preslia.* 1970;42(3):220–224. Reference Source

[ref-14] Denton A Yatsenko H Jay J : Sanger Tree of Life wet laboratory protocol collection V.1. *protocols.io.* 2023. 10.17504/protocols.io.8epv5xxy6g1b/v1

[ref-15] di Menna ME Flåøyen A Ulvund MJ : Fungi on *Narthecium ossifragum* leaves and their possible involvement in alveld disease of Norwegian lambs. *Vet Res Commun.* 1992;16(2):117–124. 10.1007/BF01839008 1496813

[ref-16] Diesh C Stevens GJ Xie P : JBrowse 2: a modular genome browser with views of synteny and structural variation. *Genome Biol.* 2023;24(1): 74. 10.1186/s13059-023-02914-z 37069644 PMC10108523

[ref-17] Doležel J Greilhuber J Suda J : Estimation of nuclear DNA content in plants using flow cytometry. *Nat Protoc.* 2007;2(9):2233–2244. 10.1038/nprot.2007.310 17853881

[ref-19] Ewels P Magnusson M Lundin S : MultiQC: summarize analysis results for multiple tools and samples in a single report. *Bioinformatics.* 2016;32(19):3047–3048. 10.1093/bioinformatics/btw354 27312411 PMC5039924

[ref-18] Ewels PA Peltzer A Fillinger S : The nf-core framework for community-curated bioinformatics pipelines. *Nat Biotechnol.* 2020;38(3):276–278. 10.1038/s41587-020-0439-x 32055031

[ref-20] Flåøyen A Bratberg B Frøslie A : Nephrotoxicity and hepatotoxicity in calves apparently caused by experimental feeding with *Narthecium ossifragum*. *Vet Res Commun.* 1995;19(1):63–73. 10.1007/BF01839253 7762142

[ref-21] Formenti G Abueg L Brajuka A : Gfastats: conversion, evaluation and manipulation of genome sequences using assembly graphs. *Bioinformatics.* 2022;38(17):4214–4216. 10.1093/bioinformatics/btac460 35799367 PMC9438950

[ref-22] Grüning B Dale R Sjödin A : Bioconda: sustainable and comprehensive software distribution for the life sciences. *Nat Methods.* 2018;15(7):475–476. 10.1038/s41592-018-0046-7 29967506 PMC11070151

[ref-23] Hagerup O : Pollination in the Faroes—in spite of rain and poverty in insects. *Det Kongelige Danske Videnskabernes Selskab: Biologiske Meddelelser.* 1951;18:1–48. Reference Source

[ref-24] Hanson L Brown RL Boyd A : First nuclear DNA C-values for 28 angiosperm genera. *Ann Bot.* 2003;91(1):31–38. 10.1093/aob/mcg005 12495917 PMC4240350

[ref-25] Harry E : PretextView (Paired REad TEXTure Viewer): a desktop application for viewing pretext contact maps.2022. Reference Source

[ref-26] Howe K Chow W Collins J : Significantly improving the quality of genome assemblies through curation. *GigaScience.* 2021;10(1): giaa153. 10.1093/gigascience/giaa153 33420778 PMC7794651

[ref-27] Jacquemart AL : Selfing in *Narthecium ossifragum (Melanthiaceae)*. *Plant Sys Evol.* 1996;203:99–110. 10.1007/BF00985240

[ref-28] Jay J Yatsenko H Narváez-Gómez JP : Sanger Tree of Life sample preparation: triage and dissection. *protocols.io.* 2023. 10.17504/protocols.io.x54v9prmqg3e/v1

[ref-29] Kerpedjiev P Abdennur N Lekschas F : HiGlass: web-based visual exploration and analysis of genome interaction maps. *Genome Biol.* 2018;19(1): 125. 10.1186/s13059-018-1486-1 30143029 PMC6109259

[ref-30] Kurtzer GM Sochat V Bauer MW : Singularity: scientific containers for mobility of compute. *PLoS One.* 2017;12(5): e0177459. 10.1371/journal.pone.0177459 28494014 PMC5426675

[ref-31] Lawniczak MKN Davey RP Rajan J : Specimen and sample metadata standards for biodiversity genomics: a proposal from the Darwin Tree of Life project [version 1; peer review: 2 approved with reservations]. *Wellcome Open Res.* 2022;7:187. 10.12688/wellcomeopenres.17605.1

[ref-32] Li H : Minimap2: pairwise alignment for nucleotide sequences. *Bioinformatics.* 2018;34(18):3094–3100. 10.1093/bioinformatics/bty191 29750242 PMC6137996

[ref-33] Loureiro J Rodriguez E Dolezel J : Two new nuclear isolation buffers for plant DNA flow cytometry: a test with 37 species. *Ann Bot.* 2007;100(4):875–888. 10.1093/aob/mcm152 17684025 PMC2749623

[ref-34] Löve A Löve D : Chromosome numbers of central and northwest European plant species.Stockholm: Almqvist & Wiksell,1961;5.

[ref-35] Malmer N : Studies on mire vegetation in the Archaean area of south-western Götaland (South Sweden). I. Vegetation and habitat conditions on the Akhult mire.Stockholm: Almqvist & Wiksell,1962;7. Reference Source

[ref-36] Manni M Berkeley MR Seppey M : BUSCO update: novel and streamlined workflows along with broader and deeper phylogenetic coverage for scoring of eukaryotic, prokaryotic, and viral genomes. *Mol Biol Evol.* 2021;38(10):4647–4654. 10.1093/molbev/msab199 34320186 PMC8476166

[ref-37] Merkel D : Docker: lightweight Linux containers for consistent development and deployment. *Linux J.* 2014;2014(239): 2, [Accessed 2 April 2024]. Reference Source

[ref-38] Narváez-Gómez JP Mbye H Oatley G : Sanger Tree of Life sample homogenisation: Covaris cryoPREP ^®^ automated dry pulverizer V.1. *protocols.io.* 2023. 10.17504/protocols.io.eq2lyjp5qlx9/v1

[ref-39] Oatley G Sampaio F Kitchin L : Sanger Tree of Life HMW DNA Fragmentation: Covaris g-TUBE for ULI PacBio. *protocols.io.* 2023; [Accessed 13 June 2024]. 10.17504/protocols.io.q26g7pm81gwz/v1

[ref-40] Paulli S : A Quadripartitum botanicum de simplicum medicamentorum.Argentorati: Impensis Fil. Simonis Paulli,1667.

[ref-41] Pellicer J Powell RF Leitch IJ : The application of flow cytometry for estimating genome size, ploidy level endopolyploidy, and reproductive modes in plants.In: Besse, P. (ed.) *Methods Mol Biol. (Clifton, N.J.).*New York, NY: Humana,2021;2222:325–361. 10.1007/978-1-0716-0997-2_17 33301101

[ref-42] Pointon DL Eagles W Sims Y : sanger-tol/treeval v1.0.0 – Ancient Atlantis. 2023. 10.5281/zenodo.10047654

[ref-43] Pollock ML Wishart H Holland JP : Photosensitisation of livestock grazing *Narthecium ossifragum*: current knowledge and future directions. *Vet J.* 2015;206(3):275–283. 10.1016/j.tvjl.2015.07.022 26324639

[ref-44] Quinlan AR Hall IM : BEDTools: a flexible suite of utilities for comparing genomic features. *Bioinformatics.* 2010;26(6):841–842. 10.1093/bioinformatics/btq033 20110278 PMC2832824

[ref-45] Ranallo-Benavidez TR Jaron KS Schatz MC : GenomeScope 2.0 and Smudgeplot for reference-free profiling of polyploid genomes. *Nat Commun.* 2020;11(1): 1432. 10.1038/s41467-020-14998-3 32188846 PMC7080791

[ref-46] Rao SSP Huntley MH Durand NC : A 3D map of the human genome at kilobase resolution reveals principles of chromatin looping. *Cell.* 2014;159(7):1665–1680. 10.1016/j.cell.2014.11.021 25497547 PMC5635824

[ref-47] Ratnasingham S Hebert PDN : bold: the Barcode of Life Data system ( http://www.barcodinglife.org). *Mol Ecol Notes.* 2007;7(3):355–364. 10.1111/j.1471-8286.2007.01678.x 18784790 PMC1890991

[ref-48] Rhie A McCarthy SA Fedrigo O : Towards complete and error-free genome assemblies of all vertebrate species. *Nature.* 2021;592(7856):737–746. 10.1038/s41586-021-03451-0 33911273 PMC8081667

[ref-49] Rhie A Walenz BP Koren S : Merqury: reference-free quality, completeness, and phasing assessment for genome assemblies. *Genome Biol.* 2020;21(1): 245. 10.1186/s13059-020-02134-9 32928274 PMC7488777

[ref-50] Strickland M Cornwell C Howard C : Sanger Tree of Life fragmented DNA clean up: manual SPRI. *protocols.io.* 2023. 10.17504/protocols.io.kxygx3y1dg8j/v1

[ref-51] Summerfield RJ : *Narthecium ossifragum* (L.) Huds. *J Ecol.* 1974;62(1):325–339. 10.2307/2258895

[ref-52] Todorovic M Oatley G Howard C : Sanger Tree of Life HMW DNA extraction: automated plant MagAttract v.2. *protocols.io.* 2023. 10.17504/protocols.io.36wgq3n13lk5/v1

[ref-53] Tønnesen HH Mysterud I Karlsen J : Identification of singlet oxygen photosensitizers in lambs drinking water in an alveld risk area in West Norway. *J Photochem Photobiol B.* 2013;119:37–45. 10.1016/j.jphotobiol.2012.12.003 23313826

[ref-54] Tsaliki M Diekmann M : Fitness and survival in fragmented populations of *Narthecium ossifragum* at the species’ range margin. *Acta Oecologica.* 2009;35(3):415–421. 10.1016/j.actao.2009.01.008

[ref-55] Tutin TG Heywood VH Burges NA : Flora Europaea. Cambridge, UK: Cambridge University Press,1980;5. Reference Source

[ref-56] Twyford AD Beasley J Barnes I : A DNA barcoding framework for taxonomic verification in the Darwin Tree of Life project [version 1; peer review: 2 approved]. *Wellcome Open Res.* 2024;9:339. 10.12688/wellcomeopenres.21143.1 39386966 PMC11462125

[ref-57] Uliano-Silva M Ferreira JGRN Krasheninnikova K : MitoHiFi: a python pipeline for mitochondrial genome assembly from PacBio high fidelity reads. *BMC Bioinformatics.* 2023;24(1): 288. 10.1186/s12859-023-05385-y 37464285 PMC10354987

[ref-58] Ulvund MJ : Important sheep flock health issues in Scandinavia/northern Europe. *Small Ruminant Research.* 2012;106(1):6–10. 10.1016/j.smallrumres.2012.04.011

[ref-59] Vasimuddin M Misra S Li H : Efficient architecture-aware acceleration of BWA-MEM for multicore systems.In: *2019 IEEE International Parallel and Distributed Processing Symposium (IPDPS).*IEEE,2019;314–324. 10.1109/IPDPS.2019.00041

[ref-60] Wisløff H Flåøyen A Ottesen N : *Narthecium ossifragum* (L.) Huds. causes kidney damage in goats: morphologic and functional effects. *Vet Pathol.* 2003;40(3):317–327. 10.1354/vp.40-3-317 12724574

[ref-61] Zhou C : c-zhou/oatk: Oatk-0.1.2023. 10.5281/zenodo.7631375

[ref-62] Zhou C McCarthy SA Durbin R : YaHS: yet another Hi-C scaffolding tool. *Bioinformatics.* 2023;39(1): btac808. 10.1093/bioinformatics/btac808 36525368 PMC9848053

