# Supplementary material for: The genome sequence of the Bog Asphodel, Narthecium ossifragum (L.) Huds
Source: Wellcome Open Res. 2025 Apr 14;10:194. [Version 1] doi: 10.12688/wellcomeopenres.24049.1 (PMC12351241; doi:10.12688/wellcomeopenres.24049.1)
Supplement: Peer review history

# Reviewer report: Reviewer response for version 1

**DOI:** 10.21956/wellcomeopenres.26533.r125694

**Published:** 2025-08-13

**Authors:** David P Horvath

**Affiliations:** 1 USDA-Agricultural Research Service, Fargo, USA

This manuscript is from the same group that submitted the last one, and contains all of the same analyses and this time. The group has fixed all the issues I had with their previous work, and this paper is ”acceptable”  as is. There are a few minor notes in the attached marked-up file.

Are sufficient details of methods and materials provided to allow replication by others?

Yes

Is the rationale for creating the dataset(s) clearly described?

Yes

Are the datasets clearly presented in a useable and accessible format?

Yes

Are the protocols appropriate and is the work technically sound?

Yes

Reviewer Expertise:

Plant stress physiology, signaling, development, transcriptomics, and genomics.

I confirm that I have read this submission and believe that I have an appropriate level of expertise to confirm that it is of an acceptable scientific standard.

# Reviewer report: Reviewer response for version 1

**DOI:** 10.21956/wellcomeopenres.26533.r122679

**Published:** 2025-05-27

**Authors:** Peng-Fei Ma

**Affiliations:** 1 Chinese Academy of Sciences, Kunming, China

In this study, the nuclear genome of *Narthecium ossifragum* was sequenced and assembled with the third-generation sequencing. The genomic data  presented will be helpful for understanding the evolution of this species and the whole lineage it represented. The quality of assembled genome is generally good but I am concerned about one thing. There is a relatively big gap between the estimated genome size (490 Mb by flow cytometry) and assembled genome size (378 Mb). How to explain that?

Are sufficient details of methods and materials provided to allow replication by others?

Yes

Is the rationale for creating the dataset(s) clearly described?

Yes

Are the datasets clearly presented in a useable and accessible format?

Yes

Are the protocols appropriate and is the work technically sound?

Yes

Reviewer Expertise:

Plant genomics and evolution.

I confirm that I have read this submission and believe that I have an appropriate level of expertise to confirm that it is of an acceptable scientific standard.
